# Role of Neuroinflammation in Autism Spectrum Disorder and the Emergence of Brain Histaminergic System. Lessons Also for BPSD?

**DOI:** 10.3389/fphar.2020.00886

**Published:** 2020-06-16

**Authors:** Nermin Eissa, Adel Sadeq, Astrid Sasse, Bassem Sadek

**Affiliations:** ^1^Department of Pharmacology and Therapeutics, College of Medicine & Health Sciences, United Arab Emirates University, Al Ain, United Arab Emirates; ^2^Zayed Center for Health Sciences, United Arab Emirates University, Al Ain, United Arab Emirates; ^3^College of Pharmacy, Al Ain University of Science and Technology, Al Ain, United Arab Emirates; ^4^School of Pharmacy and Pharmaceutical Sciences, Trinity College Dublin, University of Dublin, Dublin, Ireland

**Keywords:** behavioral and psychological symptoms of dementia, Alzheimer’s disease, schizophrenia, autism spectrum disorder, cytokines, neuroinflammation, central histamine receptors, H3R antagonists

## Abstract

Many behavioral and psychological symptoms of dementia (BPSD) share similarities in executive functioning and communication deficits with those described in several neuropsychiatric disorders, including Alzheimer’s disease (AD), epilepsy, schizophrenia (SCH), and autism spectrum disorder (ASD). Numerous studies over the last four decades have documented altered neuroinflammation among individuals diagnosed with ASD. The purpose of this review is to examine the hypothesis that central histamine (HA) plays a significant role in the regulation of neuroinflammatory processes of microglia functions in numerous neuropsychiatric diseases, i.e., ASD, AD, SCH, and BPSD. In addition, this review summarizes the latest preclinical and clinical results that support the relevance of histamine H1-, H2-, and H3-receptor antagonists for the potential clinical use in ASD, SCH, AD, epilepsy, and BPSD, based on the substantial symptomatic overlap between these disorders with regards to cognitive dysfunction. The review focuses on the histaminergic neurotransmission as relevant in these brain disorders, as well as the effects of a variety of H3R antagonists in animal models and in clinical studies.

## Introduction

### ASD as a Prototype for Neuropsychiatric Disorders

Alzheimer’s disease (AD) patients are often found to show apathy, depression, eating, and sleeping disorders, aggressive behavior, as well as other non-cognitive symptoms (Selles et al., 2018). These symptoms are usually associated with AD pathology but are often neglected as part of disease progression due to the early and more profound disturbances of memory centers in the hippocampus and entorhinal cortex. AD comprises up to 80% of all dementias. Behavioral and psychological symptoms of dementia (BPSD) in AD are known recently to correlate with gray matter (GM) atrophy and, also with white matter (WM) damage. WM damage and its relationship with GM atrophy are reported in AD (Makovac et al., 2016). Additionally, Sokol et al. reported that Amyloid-β protein precursor (βAPP) and its metabolites to be dysregulated not only in AD, but also in Autism spectrum disorder (ASD), and that the secreted variant of APP may lead to increased brain WM. WM structure is dynamic and is essential to cognitive function (Courchesne et al., 2003; Dawson et al., 2007). WM is largely composed of glia including microglia and it was proposed that neuroinflammation along with increased myelination, may contribute more to the WM enlargement in ASD (Herbert, 2005; Fields, 2008; Aoki et al., 2017; Sokol et al., 2019). Neuroinflammation appears to be similar in ASD and AD (Herbert, 2005), hence, applying known pathways in AD to ASD as proposed, should provide drug targets for ASD. Therefore, knowledge from better developed field as AD opens the door to better understand ASD.

Interestingly, BPSD are present in almost 90% of patients diagnosed with AD, characterized as a disorder of heterogeneous degenerative symptoms with memory and cognitive deficits considered as the core symptoms across multiple symptom domains (Chakraborty et al., 2019). Many BPSD share similarities with symptoms observed in AD, schizophrenia (SCH), and ASD including depression, anxiety, executive functioning deficits, and communication deficits (Wallace et al., 2016; Rhodus et al., 2019). ASD is a biologically based persistent neurodevelopmental disorder of which the core symptoms include impaired social interaction and repetitive behaviors with restricted interests (Baronio et al., 2015). The term ASD became much more used in the medical literature with the publishing of the Diagnostic and Statistical Manual of Mental Disorders (DSM-5). The history of the concept of ASD was rather well described by Ousley and Cermak (Ousley and Cermak, 2014). The core symptoms of ASD, e.g., stereotypy, repetitive behavior, and restricted interests, can be typically diagnosed in early developmental period in childhood that are persistent for the whole lifetime (Andres, 2002). The incidence of ASD has been reported to be increasing, which attracted the attention of the public but also scientists (Sheldrick and Carter, 2018; Xu et al., 2018). As estimated worldwide, prevalence of patients with ASD diagnosis is remarkably high. Current prevalence for ASD is approximately one in 160 children worldwide, and is expected to rise (Arvidsson et al., 2018). Despite the high prevalence rate, the etiology and pathogenesis of this disorder are still largely unknown and remain a matter of speculation. The lack of a specific etiologic diagnosis can be attributed to limited human brain accessibility and the complexity of the neurobiology of its activity (Nestler and Hyman, 2010). ASD is a heterogeneous group of neurobehavioral abnormalities with different recognized genetic and environmental origins. Genetic and environmental factors are strongly suggested to be involved in incidence of ASD (Baronio et al., 2015). Additionally, the heterogeneous behavioral symptoms and neuropsychiatric comorbidities in autistic children make it difficult to decipher the pathophysiology of this disorder, and consequently to develop a fundamental therapeutic approach to ASD. Therefore, subgrouping of ASD children with shared symptoms and shared molecular changes into several categories and observing their response to intervention is essential (James et al., 2009). Pharmacological treatments addressing core symptoms in ASD still remain challenging. Despite expanding awareness and advances in early age, efficacious reversal of these persistent autistic symptoms is not yet achieved. To date, risperidone and aripiprazole are the only two ASD-specific drugs approved by the US Food and Drug Administration (FDA) for improving behavioral ASD associated symptoms, such as irritability (Matson et al., 2011). There is lack of effective therapeutic interventions that address ASD hallmark symptoms (Sheldrick and Carter, 2018; Xu et al., 2018). Pharmacotherapeutic options that are currently used target accompanying symptoms in ASD, but are not disease-modifying and do not provide symptomatic control of core symptoms (Wong and Smith, 2006; Hanson et al., 2007). These accompanying physiological and psychiatric symptoms of ASD include attention deficit, anxiety, irritability, hyperactivity, self-injuries, aggression, in addition to sleep, sensory, and gastrointestinal disturbances (Lai et al., 2014; Summers et al., 2017). Psychiatric drugs are frequently used for treating these symptoms in autistic children (Findling, 2005). Despite the outstanding research that has been accomplished on ASD, complete and effective treatments targeting ASD core symptoms has been challenging and not yet achieved, as mentioned earlier. Therefore, significant progress toward the goal of identifying treatments for improving and potentially even curing core symptoms of ASD is of high importance, aiming to provide better quality of life for the suffering individuals and relieving the burden on their families. This heterogeneity may be due to the display of a wide spectrum of symptoms. The risk architecture of ASD included both genetic as well as environmental factors, however, there is not any unifying genetic or environmental factor linked to this disorder (Hassan and Mokhtar, 2019) (Stubbs et al., 2016). Also, the variety of interactions between genes, epigenetics, and the exposure to environmental factors all play critical and definite roles in developing ASD (Muhle et al., 2004). The risk of developing ASD was reported to be 35–40% due to genetic variability and around 60% due to pre,- peri-, and postnatal environmental factors (Hallmayer et al., 2011). Accordingly, environmental factors in terms of ASD risk included prenatal and perinatal complications (Glasson et al., 2004; Maramara et al., 2014), birth and neonatal complications (Gardener et al., 2011; Guinchat et al., 2012), advanced parental age, assisted reproductive technologies, nutritional factors, maternal viral infection, autoimmune diseases, and exposure to environmental chemicals, toxins, and medications such as the anticonvulsant valproic acid (VPA) (Kern and Jones, 2006; Kolevzon et al., 2007; Emberti Gialloreti et al., 2019). Therefore, a better understanding of gene-environmental interplay in the pathogenesis of ASD may explain better the pathophysiology of ASD, hence lead to an optimized therapeutic strategy.

### Common Neurotransmitter Changes in ASD, BPSD, and SCH

The reported interplay between ASD and late life dementia highlights shared neuroanatomic areas between ASD and late life dementias, that could help to provide valuable insights for the development of therapeutic strategies for both ASD and behavioral features seen in mild cognitive impairments (MCIs) and states of dementia (Crawford et al., 2014). Recognition of possible relationships between clinical features of dementia and ASD has sparked a recent scientific research. Along with the genetic factors and environmental influences, growing evidences suggested an association between the onset and progression of ASD and a variety of brain neurotransmitter systems such as acetylcholine (ACh), dopamine, serotonin, glutamate, γ-amino butyric acid, and histamine (HA) (Shah and Wing, 2006; Bacchelli et al., 2015; Ellenbroek and Ghiabi, 2015; Wang et al., 2015; Chen et al., 2017; Hellings et al., 2017; Hellmer and Nystrom, 2017; Naaijen et al., 2017; Nakai et al., 2017; Paval, 2017; Paval et al., 2017). Histaminergic and cholinergic altered neurotransmission are thought to play a crucial role in the ASD-related behavioral phenotype (Karvat and Kimchi, 2014; Baronio et al., 2015; Wright et al., 2017). Previous reports suggested that an impaired cholinergic system causes cognitive problems that may include social problems, which were reversed by donepezil treatments, an acetylcholinesterase inhibitor (ACEI) (Riedel et al., 2009; Karvat and Kimchi, 2014). Mounting evidence from preclinical studies indicated notably that H3R antagonists/inverse agonists exhibited cognition-enhancing properties (Witkin and Nelson, 2004; Passani and Blandina, 2011; Sadek et al., 2016b; Sadek and Stark, 2016). Both AChE and histamine H3 receptors (H3R) auto- and heteroreceptors are suggested to be involved in the modulation of several central neurotransmitters, including ACh and HA, which are associated with cognition. Moreover, BPSD represent a heterogeneous group of neuropsychiatric and behavior symptoms occurring in patients with dementia, and are clinically relevant as cognitive symptoms which correlate strongly to the degree of functional and cognitive impairment (Cerejeira et al., 2012). Furthermore, it has been revealed that several brain neurotransmitters are involved in a particular behavioral syndrome of BPSD and ASD. The imbalances of different neurotransmitters and their role in BPSD clinical manifestation have been extensively investigated. Findings of recent trials of ACEIs, supported that this class of drugs may be effective in managing BPSD (Lanari et al., 2006). Dementia is a consequence of neurodegeneration in brain, and AD is the most common form of dementia which is characterized by progressive cognitive and behavioral impairments (Dillon et al., 2013). The cholinergic neurotransmitter system has long been known to have an important role in the cognitive decline and memory deficits of AD (Sultzer, 2018). This view supports the recent findings of promising improvements in BPSD by ACEIs, highlighting the significant role of ACh in enhancing not only cognition and memory but also behavioral symptoms. Moreover, social functioning impairment common in ASD and SCH may be due to underlying mechanisms such as deficits in theory of mind (ToM), that are common in both disorders, as both overlap genetically and symptomatically (De Crescenzo et al., 2019). Lack of ToM skills has been also proposed to be an important part of AD. ToM refers to the ability of an individual to understand the mental states of oneself and others, and depends on executive functions and memory (Castelli et al., 2011). It was reported that 65% of AD dementia patients exhibited cognitive ToM deficits, and these deficits were associate with multiple domains of cognitive impairments (Yildirim et al., 2020). Similar to ACEIs, H3R antagonists are reported to have cognitive enhancing effects with positive results in memory and attention (Nathan et al., 2013), suggesting the important role of histamine in disorders associated with memory and cognitive impairments, and proposing the special role it might have in ToM. In addition, Passani et al. reported preclinically, that several neurotransmitters including histamine regulated social recognition and memory consolidation in amygdala and hippocampus (Passani et al., 2017). In line with these findings, the significance of this research area to disclose the etiology of ASD and BPSD is substantially important, for developing novel agents with multiple pharmacological effects for treatment of neuropsychiatric disorders of a multifactorial nature, such as ASD.

## Similarities Between ASD and Other Neuropsychiatric Disorders Including BPSD

ASD, SCH, and BPSD are all significant public health problems. Scientists have recently explored the association between ASD and SCH, but the outcomes are inconsistent (Zheng et al., 2018). The relationship between ASD and SCH is complex and has experienced significant reconsiderations over the past seven decades. In the mid-twentieth century, the two neuropsychiatric disorders were in fact regarded as being one condition, however, from the early 1970s, the two began to be looked at as separate conditions. Subsequently, the separation of the two disorders was justified, with the age at onset being the most evident example where the disorders differ. However, it is now widely recognized that there is substantial overlap between the two conditions, based on genetic underpinnings, epidemiological similarities, and the high rates of co-occurrence (Wood, 2017).

Interestingly, behavioral characteristics of ASD have been described in individuals with MCI or early dementia, demonstrating the possibility of late-life emergence of behaviors characteristic of ASD as part of MCI or AD (Rhodus et al., 2019) (**Table 1**). Moreover, the genetic basis of ASD and AD implies common associations like memory deficits, cognition changes, demyelination, oxidative stress and inflammation, a fundamental part of both disorders (**Table 1**) (Khan et al., 2016). Involvement of microglial function is increasingly recognized in the mechanism of AD and has been discussed in relation to BPSD, although there is a debate whether glial activation is cause or consequence of AD, or even a protective response (Selles et al., 2018). The similarities between ASD and BPSD as well as the common mechanisms of ASD and AD are summarized in **Table 1**.

**Table 1:** Relationship between ASD, BPSD and AD.

Diagnostic criteria for ASD in the Diagnostic and Statistical Manual of Mental Disorders (Rhodus et al., 2019)	Similarities of ASD and BPSD (Rhodus et al., 2019)	Common mechanisms of ASD and AD (Khan et al., 2016)
- Deficits in social communication and social interaction - Restricted, repetitive patterns of behavior, interests, or activities including repetitive movements, use of objects, or speech - Inflexibility in terms of routines	- Anxiety - Depression - Executive functioning deficits - Communication deficits	- Inflammation - Oxidative Stress - Synapse formation - Myelination - Methylation - Impaired cholinergic system

## Neuroinflammation in ASD and Comparison With Other Neurocognitive Disorders

Neuroinflammation is a response that involves neurons, microglia and macroglia, which are cells that are present in the central nervous system (CNS) (Bradl and Hohlfeld, 2003; Carson et al., 2006a). Neuroinflammation has been reported to characterize many neurodegenerative diseases and neuropsychiatric conditions such as multiple sclerosis, narcolepsy, AD, Parkinson’s disease (PD), and ASD (Carson et al., 2006b; Frick et al., 2016). Autistic individuals often show signs of altered inflammatory responses and neuro-immune system abnormalities throughout life, which implicates a potential role of inflammation in the etiology of ASD. This is further confirmed by increasing clinical and experimental evidence that links altered immune and inflammatory responses with the pathogenesis of ASD (Lucchina and Depino, 2014). Moreover, post mortem studies have supported this hypothesis, documenting substantial neuroinflammation in several brain regions of patients with ASD (Vargas et al., 2005).

**Figure 1:**
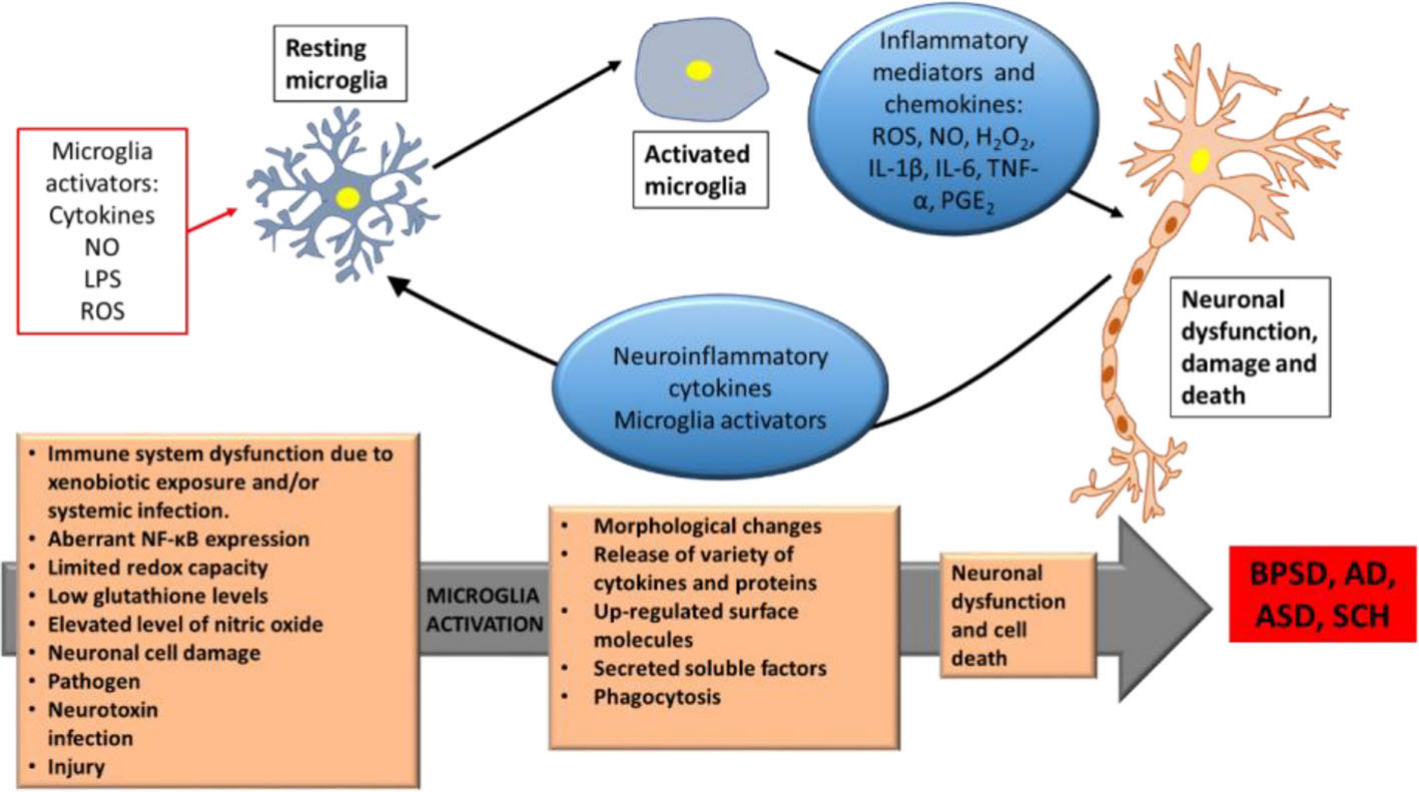
Schematic depiction of microglia activation neuronal cell death in BPSD, AD, ASD, and SCH. Neuroinflammatory proteins and cytokines due to microglia activation by genetic and different environmental activators, leading to neuron dysfunctions and cell death. BPSD, Behavioral and Psychological Symptoms of Dementia; AD, Alzheimer’s disease; ASD, Autism Spectrum Disorder; SCH, Schizophrenia; NO, Nitric Oxide; LPS, Lipopolysaccharide; ROS, Reactive Oxygen Species; H_2_O_2_, hydrogen peroxide; IL-1β, Interleukin-1β; IL-6, Interleukin-6; TNF-α, tumor necrosis factor-α; PGE_2_, Prostaglandin E2; NF-κB, Nuclear Factor kappa-light-chain-enhancer of activated B cells. Modified after (Shabab et al., 2017).

Mounting evidences supported a link between inflammation and neuropsychiatric disorders. ASD and SCH share several behavioral symptoms that might reflect the same biological basis, including inflammation. Both disorders share impairments in social communications and some degree of genetic overlap (Prata et al., 2017). Delusions and hallucinations represent the positive symptoms of SCH, while autistic traits are features of negative symptoms of SCH, that include motivational deficits, social withdrawal, poverty of speech, diminished emotional reactivity, and psychomotor expression (Kirkpatrick et al., 2006; Strauss et al., 2013; Harvey et al., 2019). Recently, Goldsmith et al. reported the associations between inflammatory markers and negative symptoms of SCH, and that inflammation is one mechanism that may underlie these negative symptoms (Goldsmith and Rapaport, 2020). Since SCH and ASD have been associated with chronic and low-grade inflammatory states, hence, a considerable number of pro-inflammatory biomarkers, including cytokines such as IL-6, TNF-α, IL-1β, IL-8, IFN-γ, have been identified in both, suggesting the related symptomatic overlap (Cox et al., 2015; Lv et al., 2015; Masi et al., 2015). Microglia, the brain’s resident inflammatory cells, have a critical role in mediating neuroinflammation and regulating brain development and homeostasis. In fact, they play a critical role in defence and tissue repair. Microglia activation is the first sign of neuroinflammation, and abnormalities in microglia have been implicated in autism (Carson et al., 2006a; Frick et al., 2016). When being activated, microglia may cause a neuronal dysfunction and cell death (neurodegenerative role). Some of the biological effects and consequences of activated microglia include rounding-up, proliferation, migration, phagocytosis, presentation of antigens to T-cells, release of a variety of oxidants such as reactive oxygen species, and activation of several genes and proteins, such as inducible nitric oxide synthase (iNOS), cyclooxygenase 1 (COX1), cyclooxygenase 2 (COX2), and a variety of proinflammatory cytokines including interleukin-1β (IL-1β), tumor necrosis factor alpha (TNF-α) (**Figure 1**). Notably, these effects are also observed in autism (Monnet-Tschudi et al., 2011). Chronic or excessive neuroinflammation has been diagnosed in ASD (Kern et al., 2015), this observed chronic glia activation and altered inflammatory function may be partly responsible for the behavioral features in ASD, as chronic peripheral inflammation and abnormal inflammatory responses in the brain may lead to cognitive dysfunction (Lucchina and Depino, 2014).

**Figure 2:**
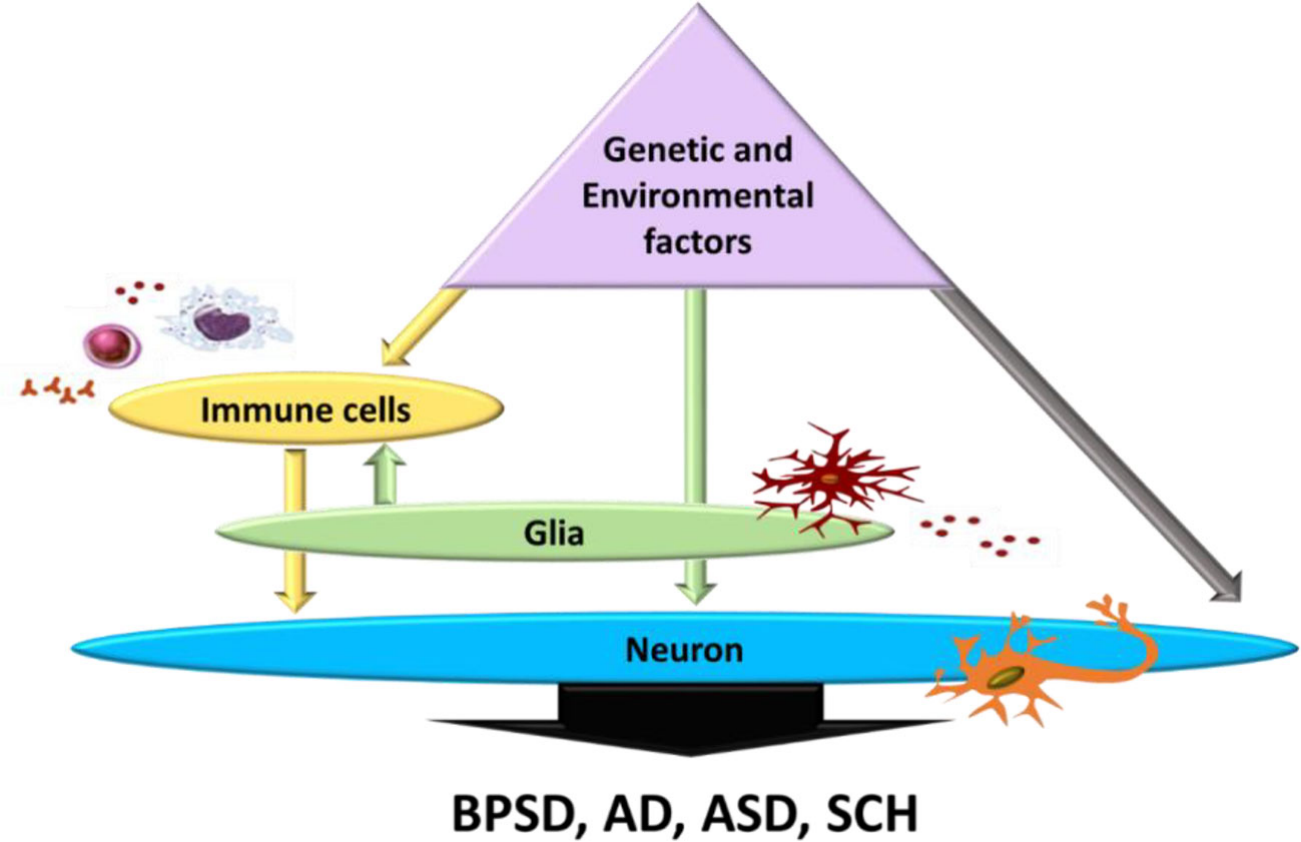
Effect of genetic and environmental factors on neuronal dysfunction and immune response modulating BPSD, AD, ASD, and SCH symptoms. All possibilities contributing to ASD through glia activation (grey arrow), or through directly altering peripheral immune cells (white arrows) which in turn activates glia affecting the neuronal function (black arrows). BPSD, Behavioral and Psychological Symptoms of Dementia; AD, Alzheimer’s disease; ASD, Autism Spectrum Disorder; SCH, Schizophrenia. Adapted from (Depino, 2013).

During pregnancy, both environmental and genetic risk factors may affect inflammatory response of new-borns, hence altering postnatal brain development (Adams-Chapman and Stoll, 2006). These genetic and environmental factors can directly elicit chronic neuroinflammation which in turn may modulate neuronal function and immune response *via* glia activation, or directly by affecting neuronal function (Depino, 2013) (**Figure 2**). Valproic acid (VPA), as an environmental risk factor, elicited activation in different brain regions, with evidence of long-lasting glia activation in the hippocampus and the cerebellum (Lucchina and Depino, 2014). The hippocampus (Depino et al., 2011) and cerebellum (DeLorey et al., 2008; Martin et al., 2010) are two brain regions linked to autism-related behavior, namely, limited social interaction and repetitive behaviors. Additionally, several studies showed that altered social behavior in adult mice may be due to cerebellar inflammation as the cerebellum is considered to be involved in executive and cognitive functions (Shi et al., 2009; Koziol et al., 2014; Lucchina and Depino, 2014; Wang et al., 2014). Furthermore, evidences suggested that astrocyte and microglia activation in the cortex and cerebellum increase expression of cytokines, including IL-6, TNF-α, MCP-1, TGF-β1, IFN-λ, interferon gamma, IL-8, and other associated genes involved with the immune response in different brain regions of autistic subjects (Vargas et al., 2005; Chez et al., 2007; Garbett et al., 2008; Li et al., 2009; Chez and Guido-Estrada, 2010). Alternatively, both these environmental and genetic factors could chronically alter immune response through increasing production of free radicals, which consequently activate glia cells, increasing the inflammatory response and then affecting neurons, thus mediating clinical symptoms of autism (Depino, 2013) (**Figure 2**). These findings suggest that neuroinflammation may contribute to ASD behavioral effect, hence, controlling microglia activation and inhibiting cytokine and free radical production might be a therapeutic strategy for treating ASD. Moreover, exploration of mechanisms involved in neuroinflammation, immune-mediated pathways and targeting their modulation as a strategy for disease-modifying treatment, are promising research approaches in neurodegenerative diseases such as AD and BPSD, where the memory and cognitive deficit domain are the most prominent across several symptom domains (Chakraborty et al., 2019). AD is characterized by neuroinflammatory processes in which microglia are over-activated, resulting in the elevated production of pro-inflammatory cytokines. Increased expression of IL-1 has been reported in AD brain where several variants in genes of IL-1A and IL-1B have been found to influence AD risk. Increased IL-6 expression has been identified in AD patients, both in the periphery and CNS. Elevated levels of TNF-α has been also reported in AD patients (Su et al., 2016; Decourt et al., 2017). Moreover, increases in IL-1, IL-6, TNF-α, IL-8, IFN-γ, IL-4, and TGF-β have been reported in patients with SCH, and have been associated with negative symptoms (Potvin et al., 2008; Goldsmith et al., 2018; Momtazmanesh et al., 2019). All of which are inflammatory markers reported to be altered in ASD (Novellino et al., 2020). A recent clinical study suggested that the behavioral phenotype of ASD may develop as a consequence of neurodegenerative processes, since the frequency of ASD-like behaviors directly correlate with the progressing severity of cognitive impairment (Rhodus et al., 2019). In addition to this, another clinical study reported the association between ASD-linked symptoms and late-life degenerative dementia, where such symptoms are more prevalent in those with early onset dementia (Crawford et al., 2014). Multiple lines of evidence support neuroinflammation as a common feature of dementia, AD, and ASD, and suggest a central role of microglia in the progression of the disorder (Lucchina and Depino, 2014; Pasqualetti et al., 2015). A broader approach to the complexity of microglial subpopulations provides an opportunity to explore the phenotypic landscape of microglia-driven neuroinflammation in ASD, AD, and BPSD, hence, may assist in the identification of targets for therapeutic interventions.

Moreover, the amyloid hypothesis predicts clinical disease associated with amyloid-β loaded plaques resulting in brain atrophy in patients with AD.

## Implication of Histamine in Tourette’s Syndrome, SCH and ASD

Central histaminergic system (HS) was found to exhibit a critical role in cognition and sleep disorders, and has been reported to be involved in various brain disorders such as AD, SCH, drug dependence, and PD (Baronio et al., 2014; Wright et al., 2017).

In previous studies, it was reported that genetic histaminergic signaling abnormalities may lie behind some cases of rare diseases such as Tourette syndrome (TS) (Paschou et al., 2013). TS was also reported to be among the most prevalently comorbid neurodevelopmental disorders with ASD (Gillberg and Billstedt, 2000), sharing genetic risk factors (Clarke et al., 2012; Fernandez et al., 2012). Additionally, both conditions share upregulation of neuroinflammation (Muller, 2007; Kern et al., 2015; Theoharides et al., 2016), and increased microglia activation (Frick et al., 2016). HA has a remarkable role in neuroinflammation (Jutel et al., 2005; Theoharides et al., 2016), as well as microglia regulation (Ferreira et al., 2012; Dong et al., 2014; Rocha et al., 2014; Frick et al., 2016), suggesting that the HS may partly mediate these abnormalities. Also, a recent preclinical study revealed that HA induces microglia activation and the release of several proinflammatory mediators in rat brain through activation of H1- or H4Rs (Zhang et al., 2019). A study of TS reported a rare non-sense mutation in HDC, a gene encoding for the histidine decarboxylase enzyme that synthesizes HA from histidine (Karagiannidis et al., 2013). Other recent studies suggested *de novo* deletions overlap in HNMT, a gene which encodes the enzyme histamine-*N*-methyl transferase that inactivates HA (Griswold et al., 2012; Mulatinho et al., 2012). Furthermore, analysis of gene mapping within rare copy number variants in TS reported a significant overlap with those revealed in ASD, and some of them were in histamine pathways (Fernandez et al., 2012). All these findings of overlaps between the two disorders raised the possibility of the involvement of the HS in ASD. Furthermore, ASD and SCH were reported to share similar clinical symptoms and significant genetic overlap (Konstantareas and Hewitt, 2001; Carroll and Owen, 2009; Naguy and Naguy, 2018). Replicated findings suggested that SCH and ASD may share similar biological pathways, demonstrating that both conditions have a structural variant in chromosomal regions 16p11.2 (Weiss et al., 2008; McCarthy et al., 2009), 22q11.2 (Guilmatre et al., 2009; Vassos et al., 2010), 1q21 (Brunetti-Pierri et al., 2008; Stefansson et al., 2008; Ikeda et al., 2010; Mefford et al., 2010), and the gene neurexin (NRXN) and SHANK (Leblond et al., 2012; Sato et al., 2012). Other recent publications of copy number variations revealed rare variants at NRXN1 and catenin alpha3 loci suggesting a risk factor overlap with both ASD and SCH. In addition to the genetic overlap between both disorders, they also share behavioral symptoms as mentioned earlier. Social cognitive impairments are hallmark behavioral deficits in both, ASD and SCH (Couture et al., 2006; Meyer et al., 2011). Furthermore, neuroinflammation as a consequence of microglia activation plays an important role in both SCH and ASD (Nakagawa and Chiba, 2016). These findings suggest that HS dysfunction may be involved in the etiology of ASD, since both SCH and TS disorders have substantial genetic and symptomatic overlap with ASD. Therefore, the emergence that the HS may be implicated in ASD and may contribute to the core symptoms, necessitates further research to investigate what role the HS may or may not have in enhanced neuroinflammation.

### Histamine and Inflammation in Neurodegenerative Disorders

The possible implication of central HA to regulate neuroinflammation has received some scientific attention, but more recently, the fact that both the HS and microglial dysregulation are involved in a range of neurodegenerative pathologies and neurological conditions, highlighted the importance of HA in the regulation of microglia (Rocha et al., 2014). Evidence has been pointing to neuroinflammation as a triggering factor in neurodegenerative disorders and cognitive decline. In the brain, histamine can act either as neurotransmitter or as modulator of the innate immune system, hence modulating brain inflammatory responses (Saraiva et al., 2019). Several studies demonstrated the ability of histamine to counteract LPS-induced inflammation through the decrease of microglial migration, phagocytosis and ROS production induced by LPS, as well as the release of IL-1β (Ferreira et al., 2012) A recent finding reported microglial abnormalities in HDC knockout mouse, a validated model of TS (Baldan et al., 2014), which further supported the importance of understanding the role of HA in regulating microglial function, especially as TS and ASD have a high degree of overlap. An *in vitro* study demonstrated that microglia cells expressed all known HRs (Ferreira et al., 2012). Another experimental study suggested the role of HA in microglial inflammatory response modulation, demonstrating a dual role of HA in neuroinflammation regulation. Activated microglia modulate cell recruitment and proinflammatory cytokine release, such as IL-1β and TNF-α (Ferreira et al., 2012).

This evidence is complemented by a recent study demonstrating that HA reduces the proinflammatory microglia phenotype in the SOD1-G93A mouse model of Amyotrophic Lateral Sclerosis. It was reported that HA exerts its beneficial action only in inflammatory SOD1-G93A microglia, and on the other hand elicits a pro-inflammatory effect in non-transgenic cells (Apolloni et al., 2017). These findings demonstrated a different role for HA under physiological conditions and during an inflammatory response (Barata-Antunes et al., 2017). Another recent study demonstrated the dual role of HA in the modulation of microglial responses, suggesting that while histamine *per se* triggers microglia pro-inflammatory injurious phenotype, it can revert them oppositely under inflammatory challenge (Barata-Antunes et al., 2017), opening a new perspective for the therapeutic potential of HA to selectively improve inflammation-associated processes in disorders associated with microglia-derived inflammation. The role of H3R antagonists in stimulating the synthesis and release of HA in brain, as mentioned earlier, suggests that therapeutic use of H3R antagonists may ameliorate neuroinflammation and consequently, improving ASD behavioral symptoms. Moreover, the antioxidant effect of H3R antagonists, as demonstrated in a previous study strongly suggests that H3R antagonists may have therapeutic potential in the management of ASD (Mahmood et al., 2012; Mani et al., 2017). Previous studies have found elevated expression of proinflammatory molecules, including IL-1ß, TNF-α, IL-6, and TGF-ß in the autistic brain (Vargas et al., 2005; Depino, 2013; Goines and Ashwood, 2013; Deckmann et al., 2018). IL-1ß disruption was reported to have several neurological consequences relevant to ASD. It was also reported earlier that IL-6 overexpression in the mice CNS showed cognitive alterations, including avoidance behaviors (Heyser et al., 1997). Additionally, Vargas et al. found that transforming growth factor beta (TGF-β) was one of the most prevalent cytokines in brain tissues of individuals with ASD and is involved in social behavior. A previous study demonstrated that JNJ10181457, a H3R receptor inverse agonist reverted LPS-induced microglial IL-1ß, IL-6, and TNF-α expression, indicating that the compound inhibited microglial activation associated with inflammation (Iida et al., 2017). Similarly, another study showed that ciproxifan (1 mg/kg, i.p.) reduced the level of IL-1ß and IL-6 cytokines in the transgenic mouse brain of B6.129-Tg(APPSw)40Btla/J mice (Mani et al., 2017). Moreover, a recent study reported that H3R inverse agonist BF 2649, or selective H3R antagonist with partial H4R receptor agonist clobenpropit, significantly showed reduction in amyloid beta peptide (AβP) deposits along with marked reduction in neuronal or glial reactions in AβP infusion-induced brain pathology in a rat model (AD like pathology). However, clobenpropit showed superior effects than the BF2649 in this AD model. The results suggested that H3 and H4 receptor modulation may induce neuroprotective effect resulting in less deposition of the peptide and reduction in glia activation (Patnaik et al., 2018). Also, earlier preclinical findings showed that activation of brain histaminergic neurotransmission may be a mechanism for cognitive effects of memantine, an NMDA-receptor antagonist widely used for the treatment of AD (Motawaj et al., 2011), demonstrating the role of neurotransmission to NMDA receptors and in BPSD (Lin and Lane, 2019). Thus, these multiple lines of evidence demonstrate a strong impact of the histaminergic neurotransmission on modulation of microglia-induced neuroinflammation and associated pro-inflammatory cytokine expression. This may also suggest that cytokine imbalances could impact neural activity and mediate behavioral aspects of ASD. H3R antagonists may serve as potential therapeutics for ASD and other brain disorders with microglia-driven neuroinflammation, such as AD, SCH, and BPSD.

## Antagonists of Histamine Receptor Subtypes in ASD and Other Neuropsychiatric Disorders

Several clinical studies revealed the positive effects of H1R and H2R antagonists in children and adolescents with ASD and suffering from behavioral and sleep disturbances (Rossi et al., 1999; Linday et al., 2001). Moreover, numerous preclinical studies improved social behaviors and stereotyped repetitive behaviors of several imidazole- as well as non-imidazole–based H3R antagonists in different rodents (Witkin and Nelson, 2004; Esbenshade et al., 2008; von Coburg et al., 2009; Brown et al., 2013; Sadek et al., 2016b; Eissa et al., 2018a; Eissa et al., 2019), and are discussed below.

### H1R Antagonists

Among numerous H1R antagonists, niaprazine with noticeable sedative properties has been clinically used in subjects with behavior and sleep disorders (Rossi et al., 1999). A promising effect was found in 52% of autistic patients with associated behavior and sleep disorders, with specific efficacy on attention deficit, hyperkinesia, rigidity, hetero-aggressiveness, mild anxiety, and sleep disturbances. Rossi et al. concluded that niaprazine can be used as a first-choice drug to improve behavior and sleep disorders in patients with ASD due to its good tolerability and the presence of sedative effects. Moreover, the clinical use of the H1R antagonist cyproheptadine was reported to decrease stereotypical behaviors and to improve expressive speech in children with ASD, when compared with a group receiving haloperidol and placebo (Gudarzi et al., 2002; Akhondzadeh et al., 2004).

### H2R Antagonists

Several studies speculated that the H2R antagonist famotidine might be effective for certain ASD symptoms because it has been shown to improve certain symptoms in SCH, including improvements in eye contact avoidance, repetitive behaviors, social communication, and social interaction in children with ASD who had no history of gastrointestinal problems (Linday, 1997; Linday et al., 2001). Moreover, a very recent study showed that pretreatments of animals with famotidine prevented cell death induced by the NMDA antagonist MK-801, and therefore provided neuroprotective effects *via* modulation of the Akt/GSK-3β/β-catenin signaling pathway, an important mechanism in SCH neurobiology (Unal et al., 2019).

### H3R Antagonists

H3Rs in the CNS act as presynaptic auto- or hetero-receptors that regulate the biosynthesis and release of HA and a variety of neurotransmitters form histaminergic neurons and non-histaminergic neurons, respectively. Hence, H3Rs play a role in cognitive function and homeostatic processes, as shown in (**Table 2**). This suggests that selective and potent H3R antagonists could lead to a therapeutic approach for the improvement of cognitive decline accompanied with SCH and ASD (Witkin and Nelson, 2004; Esbenshade et al., 2008; von Coburg et al., 2009; Brown et al., 2013; Sadek et al., 2016b). To date, few studies have investigated the association of H3R antagonists and the underlying mechanism for treatment of ASD behavioral deficits (**Table 2**).

**Table 2:** Summary of H3R antagonists that have been in clinical and preclinical trials in ASD and related brain disorders.

Disorder	H3R antagonist	Clinical phase	Pharmacological effect	Reference
ASD	Ciproxifan	Preclinical	Improving some social impairments and stereotypies in mice.	(Baronio et al., 2015)
	DL77	Preclinical	Palliated sociability deficits and stereotypies.	(Eissa et al., 2018a)
	ABT-239	Preclinical	Improvement in social memory.	(Fox et al., 2005)
	E100	Preclinical	Ameliorated repetitive compulsive behaviors in a mouse model of ASD.	(Eissa et al., 2019)
ADHD	JNJ-31001074	Clinical	No significant improvements in adult patients.	(Weisler et al., 2012; Sadek et al., 2016c)
AD	ABT-288	Clinical	A randomized study did not demonstrate any significant improvements in mild to moderate AD dementia.	(Haig et al., 2014b)
	Ciproxifan	Preclinical	Improvement in increased locomotor activity in transgenic mice. Enhancement in memory deficit.	(Bardgett et al., 2011)
	GSK239512	Clinical	Positive improvement in episodic memory in patients with mild to- moderate AD. No improvement in executive function/working memory for subjects with mild to- moderate AD.	(Nathan et al., 2013; Grove et al., 2014)
	JNJ-10181457	Preclinical	Reversed scopolamine induced-cognitive deficits in rats. Regulated ACh neurotransmission.	(Galici et al., 2009)
Cognitive impairments	ABT-239	Preclinical	Attenuated scopolamine-induced deficits in cognitive tests in rodents. Improvement in social memory.	(Brown et al., 2013)
	A-431404	Preclinical	Ameliorated cognitive impairments induced by ketamine and MK-801.	(Brown et al., 2013)
	DL77	Preclinical	Improvement of cognitive deficits through different memory stages in rats.	(Eissa et al., 2018a)
	GSK189254	Preclinical	Attenuated scopolamine-induced deficits in cognitive tests in rodents.	(Ligneau et al., 2007; Medhurst et al., 2007a; Medhurst et al., 2007b; Galici et al., 2009)
	GSK207040			
	GSK334429			
	Pitolisant			
Epilepsy	DL77	Preclinical	Increased anticonvulsant activity in epilepsy models.	(Sadek et al., 2016c)
Narcolepsy	Pitolisant	Clinical	Reduced excessive daytime sleepiness.	(Baronio et al., 2014)
SCH	ABT-288	Clinical	Failed on providing cognitive improvements to patients.	(Haig et al., 2014a)
	ABT-239	Preclinical	Attenuated cognitive deficits caused by ketamine and MK-801.	(Brown et al., 2013)
	A-431404	Preclinical	Attenuated cognitive deficits caused by ketamine and MK-801.	(Brown et al., 2013)
	Ciproxifan	Preclinical	Enhancement of prepulse inhibition.	(Browman et al., 2004)
	SAR 110894	Preclinical	Normalized impaired social behavior.	(Griebel et al., 2012)
	Thioperamide	Preclinical	Enhancement of prepulse inhibition.	(Browman et al., 2004)
	Pitolisant	Preclinical	Reduced locomotor hyperactivity elicited by methamphetamine or dizolcipine. Abolished the apomorphine-induced deficit in prepulse inhibition.	(Ligneau et al., 2007)

Preclinical animal experiments have widely used thioperamide and ciproxifan which are selective and potent imidazole-based H3R antagonists (Ligneau et al., 1998; Stark et al., 2000; Brown et al., 2013). A preclinical study reported that thioperamide and ciproxifan reinforced the decreased prepulse inhibition in an animal model of SCH (Browman et al., 2004) (**Table 2**). Interestingly, H3R antagonists have shown to possess an antioxidant effect which could enhance their therapeutic use, since oxidative stress is considered to be involved in pathogenesis and pathophysiology of SCH and ASD (Mahmood et al., 2012). Moreover, considering the pro-cognitive effect of non-imidazole H3R antagonist ABT-288 in several preclinical models, a further study revealed that treatment of dysregulated cognitive function associated with SCH, the schizophrenic features remained constant for the duration of the study (Hsieh et al., 2010; Coruzzi et al., 2012).

H3R antagonist have also showed amelioration in spatial working memory deficit observed in animal model of SCH, a deficit which also characterizes patients with ASD (Steele et al., 2007). However, these initial data still need further research efforts to expand, corroborate, and achieve a better understanding of pathophysiology and therapeutic management of ASD.

Exploring the potential role of H3R antagonists in a number of CNS diseases like AD, epilepsy, attention deficit hyperactivity disorder (ADHD), narcolepsy (Witkin and Nelson, 2004; Savage et al., 2010; Kasteleijn-Nolst Trenite et al., 2013), SCH (Passani and Blandina, 2011; Baronio et al., 2015), and recently in TS (Rapanelli and Pittenger, 2016) and ASD (Baronio et al., 2015), suggest that H3R antagonists may be a potential therapeutic approach for treatment of several neurological disorders that are linked to cognitive impairment. Preclinical studies reported that ciproxifan, an imidazole-based H3R antagonist demonstrated improvements in hyperactivity and associated memory impairment after administration of this drug in a mouse model of AD (Bardgett et al., 2011). Treatment with JNJ-10181457, a selective non-imidazole H3R antagonist reversed cognitive deficits induced by scopolamine, and re-balanced the dysregulation of ACh neurotransmission (Galici et al., 2009). Impairments in cognitive functions that are commonly featured in ASD include self-regulation and social cognition. These allow people to appropriately regulate actions related to social issues and to make plans. H3R antagonists may have a potential role in rescuing such core symptoms of ASD (Heatherton and Wagner, 2011). Moreover, recent wakefulness clinical trials reported the successful effect of pitolisant (Wakix^®^), a H3R antagonist/inverse agonist marketed for the treatment of narcolepsy (Baronio et al., 2014). Pitolisant is approved by the European Medicines Agency (EMA) as well as the FDA and is the first-in-class drug to be introduced into clinics. Preclinical data suggested that pitolisant may also be a valuable drug candidate to enhance memory deficits and to treat other cognitive disorders (Ligneau et al., 2007). Pitolisant was suggested to be effective in epilepsy, which is highly comorbid with ASD (Kasteleijn-Nolst Trenite et al., 2013). Again, all these accumulated evidences support the implication of the HS in ASD. Additionally, a recent study revealed that impairments in social behavior was ameliorated by H3R antagonists in rodents exposed to phencyclidine (PCP), suggesting its therapeutic value for ASD (Griebel et al., 2012). Based on these findings, Baronio et al. assessed for the first time the effect of imidazole-based H3R antagonist ciproxifan in animal model of autism induced by maternal VPA exposure (Baronio et al., 2015) (**Table 2**). The effect of acute administration of ciproxifan (3 mg/kg) 30 min before the behavioral test demonstrated efficacy in improving some social impairments and stereotypies in VPA mice. These results suggested that some of the main clinical alterations displayed in ASD could be improved even in adulthood, as at the stage when the tests were carried out, many changes had already occurred during brain development and have reached equilibrium. Regardless, a single application of ciproxifan was effectively enough to attenuate behavioral deficit (Baronio et al., 2015). Several imidazole-based H3R antagonists, such as ciproxifan, showed potency and selectivity in preclinical animal experiments with oral bioavailability (Ligneau et al., 1998; Stark et al., 2000). However, this class of compounds appeared to have poor CNS penetration and incidences of off-target activity at other receptors including H4R were reported. In addition, imidazole-based agents showed powerful inhibition of CYP450 isoenzymes, rendering them prone to many metabolic interactions with other drugs (Berlin et al., 2011; Panula et al., 2015; Sadek and Stark, 2016). Consequently, medicinal chemistry efforts succeeded in modification of chemical structure to generate various non-imidazole H3R antagonists with higher affinity and selectivity than the imidazole-based H3R antagonist. DL77 ([1-(3-(4-tertpentylphenoxy)propyl)piperidine) is a novel non-imidazole H3R antagonist that strongly resembles the EMA and FDA approved H3R antagonist/inverse agonist pitolisant in structure (Sadek et al., 2016a). In animal studies, DL77 showed improvements in cognitive performance by exerting its action through different memory stages (**Table 2**). A very recent preclinical study demonstrated that DL77 ameliorated cognitive deficits induced by the *N*-methyl-*D*-aspartate (NMDA) receptor antagonist MK-801 in an inhibitory passive avoidance paradigm and in novel object recognition tests in rats (Eissa et al., 2018b). These findings demonstrated the potential role of DL77 for treatment of cognitive symptoms that characterize several neuropsychiatric disorders (Sadek et al., 2016c). As mentioned earlier, social cognitive deficits are hallmark characteristic of ASD (Couture et al., 2006). Preclinically, it was reported that DL77 had promising effect on sociability deficits and stereotypies in a VPA-induced mice model of ASD (Eissa et al., 2018a). Moreover, and in a recent preclinical study, the dual-active ligand E100 with high H3R antagonist affinity and balanced AChE inhibition demonstrated ameliorative effects on repetitive compulsive behaviors and neuroinflammation in a mouse model of VPA-induced ASD in mice (**Table 2**) (Eissa et al., 2019). In addition, ABT-239 showed improvement in social memory in rodents, an altered parameter in ASD (Fox et al., 2005) (**Table 2**). H3R antagonist DL77 provided promising anticonvulsant activity in experimental epilepsy models (Sadek et al., 2016c) (**Table 2**). It was also reported in a recent population study that 44% of children with ASD were subsequently diagnosed with epilepsy and 54% of children with epilepsy were subsequently diagnosed with ASD (Jokiranta et al., 2014). Several studies demonstrated the effectiveness of H3R antagonists in rescuing behavioral impairment including memory deficit in animal model of SCH (Steele et al., 2007), symptoms diagnosed also in patients of ASD. Preclinically, cognitive ameliorating effect of various non-imidazole-based H3R antagonists, as ABT-239 and A-431404 in experimental rats with cognitive impairments that is induced by ketamine and/or MK-801, demonstrated enhanced results in comparison with reference antipsychotics like risperidone or olanzapine (Brown et al., 2013). In addition to H3R antagonists enhancing effects on different cognitive domains in rodents, H3R antagonists, including ABT-239, GSK189254, GSK207040, GSK334429, and pitolisant, ameliorated scopolamine-induced deficits in cognitive tests in rodents (Fox et al., 2005; Ligneau et al., 2007; Medhurst et al., 2007a; Medhurst et al., 2007b). SAR110894, a potent H3R antagonist also showed efficacy in several animal models addressing certain aspects of cognitive impairments (Griebel et al., 2012) (**Table 2**). This suggests that H3R antagonists may be beneficial in neurological diseases that exhibit abnormalities related to the cognitive symptoms as in ASD. Considering all these evidences, BPSD as heterogeneous range of psychiatric behaviors and symptoms arising from the presence of dementia alongside with progressive decline in cognitive functions, suggests that H3R antagonists may function to improve BPSD through enhancing cognitive performance (Witkin and Nelson, 2004; Dekker et al., 2018) as in related AD, MCI, and ASD. Neurodegeneration in the brain consequently causes dementia, which develops slowly and gradually worsens over years. The cumulative evidences of H3R antagonists cognitive and memory-enhancing effects suggest its potential use in the treatment of neurodegenerative disorders such as AD (Fox et al., 2004; Alachkar et al., 2019). A preclinical research study reported that 3 weeks daily treatment of ciproxifan alleviated the hyperactivity and cognitive deficits observed in a transgenic mouse model (APP_Tg2576_) of AD. These mice exhibited formation of amyloid plaques with increasing age as well as deficits in spatial learning and memory, that was displayed in significantly greater locomotor activity and longer escape latencies in swim maze test than wild-type mice. Moreover, APP_Tg2576_ mice significant impairment in the object recognition was reversed by acute treatment with ciproxifan (3.0 mg/kg). These data support the theory that H3R antagonism may represent a pathway to cognitive enhancement and memory impairments, signifying the potential of H3R antagonist in treatment of neurodegenerative diseases, including AD (Bardgett et al., 2011; Alachkar et al., 2019) (**Table 2**). Hence, H3R antagonists may serve as a viable therapeutic strategy in the treatment of BPSD. However, the efficacy of the highly selective H3R antagonist ABT-288 across several preclinical cognitive domains was not observed clinically. In a randomized study ABT-288 failed to show efficacy in subjects with mild to moderate AD dementia (Haig et al., 2014a) (**Table 2**). In a randomized, double-blind, placebo-controlled study, investigations of H3R antagonist/inverse agonist GSK239512 to assess cognitive enhancing effects showed positive results on memory, attention (Nathan et al., 2013) and displayed improvement in episodic memory in patients with mild to moderate AD. However, it failed to show any improvements on executive function/working memory or other domains of cognition (**Table 2**) (Grove et al., 2014). On the other hand, administration of JNJ-10181457, a selective non-imidazole histamine H3R antagonist significantly reversed the cognitive deficits induced by scopolamine in rats. JNJ-10181457 also demonstrated normalization of ACh neurotransmission in the rat cortex, which indicates that selective H3R non-imidazole antagonists may be very effective in conditions with decreased levels of ACh release commonly found in cognitive disorders such as AD, dementia, ASD, and SCH. These evidences may suggest promising clinical efficacy of H3R antagonists in cognitive-related disorders, specifically those in which ACh neurotransmission is compromised (Galici et al., 2009). In accordance with this, and as several lines of scientific evidence have implicated cholinergic system abnormalities in ASD, there is substantial support for the suggestion that treatments that modulate the cholinergic system might be effective in ASD (Deutsch et al., 2010; Ghaleiha et al., 2014), including H3R antagonists. The reported H3R antagonist modulation of cholinergic system and consequent ACh release normalization suggest a potential therapeutic efficacy in BPSD, as the cholinergic dysfunction seems to play a major role in contributing to BPSD (Lanari et al., 2006). Hence, among the strategies followed for optimal management of BPSD with respect to neurochemical component, the HS approach with H3R antagonists might be promising.

## Conclusions

Current evidence from clinical and preclinical studies supports the hypothesis that the pathogenesis of ASD is linked to the exposure to inflammation at early developmental stages. The incomplete efficacy of the current therapy for ASD has driven an increased interest in developing of several approaches in searching for new prospective drugs. Clinical studies indicate that ASD children undergo chronic neuroinflammation in different brain regions involving activation of microglia. One of the therapeutic approaches to control neuroinflammation is to reduce or prevent microglial activation, and to reduce the neuro-destructive effects of chronic neuroinflammatory processes, which contributes to improved developmental outcomes. There is cumulative evidence that HA plays central roles in the CNS, both on different environmental contexts. Since all four HRs are constitutively expressed on microglia, HA has well-established role as neuron-to-glia alarm signal in the brain. Considering the dual role of HA, targeting microglial activation by modulating microglial function and suppressing the deleterious pro-inflammatory neurotoxicity maybe a valid therapeutic strategy for promoting neuroprotection and managing ASD-like behaviors. However, future research efforts are still necessary to study which exact signaling pathways and HRs are involved in this histamine-induced neuroprotective role and for better understanding of the effects of HA and/or HR ligands to inhibit neuroinflammation *in vivo* in inflammatory environments. Evidence suggests that chronic neuroinflammation may be associated with cognitive deficits, and preclinical studies indicated notably that H3R antagonists/inverse agonists have been found to exhibit mitigating effects on several neuroinflammatory processes and, also, to provide cognition-enhancing properties in preclinical animal models of ASD. Further research efforts should be conducted to develop selective H3R antagonists capable of targeting the cognitive symptoms in multifactorial disorders in the field of neuropsychiatric disorders including BPSD and ASD. Identifying neuroanatomic substrates shared between ASD and dementias might accelerate the therapy development for more than one disorder.

## Author Contributions

NE and BS: Idea, design, writing, and submission. NE, AdS, AsS and BS: Substantial contribution to the conception, formulation, and critical revision of the manuscript. All authors contributed to the article and approved the submitted version.

## Funding

The research in laboratory of BS is supported by the University Program for Advanced Research (UPAR), Center-Based Interdisciplinary Grants (31R223), and Faculty (CMHS) Grants from the Office of the Deputy Vice Chancellor of Research and Graduate Studies of United Arab Emirates University, Al Ain, United Arab Emirates.

## Conflict of Interest

The authors declare that the research was conducted in the absence of any commercial or financial relationships that could be construed as a potential conflict of interest.

## Abbreviations

ASD, Autism Spectrum Disorder; AβP, Amyloid beta peptide; ACh, Acetylcholine; AChE, Acetylcholine esterase; ACEI, Acetylcholine esterase inhibitor; AD Alzheimer’s disease; ADHD, Attention deficit hyperactivity disorder; APP, Amyloid protein precursor; BPSD, Behavioral and Psychological Symptoms of Dementia; CNS, Central Nervous System; COX, Cyclooxygenase; H3R, Histamine H3 Receptor; HA, Histamine; GSK-3β, Glycogen synthase kinase 3 beta; GM, Grey matter; DSM, Diagnostic and Statistical Manual of Mental Disorders; EMA, European Medicines Agency; FDA, Food and Drug Administration; H_2_O_2_, Hydrogen peroxide; HDC, Histidine Decarboxylase; HR, Histamine Receptor; HNMT, Histamine Nmethyl Transferase; HS, Histaminergic System; IL, Interleukin; IFN-γ, interferon gamma; LPS, Lipopolysaccharide; MCI, Mild cognitive impairment; NF-κB, Nuclear Factor kappalight-chain-enhancer of activated B cells; iNOS, Inducible nitric oxide synthase; NO, Nitric Oxide; NRXN, Neurexin; NMDA, NMethyl-D-aspartate; PCP, Phencyclidine; PD, Parkinson’s disease; PGE_2_, Prostaglandin E_2_; ROS, Reactive Oxygen Species; SCH, Schizophrenia; TNF-α, Tumor necrosis factor alpha; TS, Tourette syndrome; SOD, Superoxide dismutase 1; TGF-β, Transforming growth factor beta; ToM, Theory of Mind; VPA, Valproic Acid; i.p., Intraperitoneally; WM, White matter.
